# GSK249320, A Monoclonal Antibody Against the Axon Outgrowth Inhibition Molecule Myelin-Associated Glycoprotein, Improves Outcome of Rodents with Experimental Stroke

**Published:** 2016-11-21

**Authors:** Diana Cash, Alanna C. Easton, Michel Mesquita, John Beech, Steve Williams, Andrew Lloyd, Elaine Irving, Steven C. Cramer

**Affiliations:** 1 King’s College London, Institute of Psychiatry, UK; 2 Phastar, Chiswick, London, UK; 3 GlaxoSmithKline, Research Development, PCPS QSci, UK; 4 Department Neurology, Anatomy & Neurobiology, and Physical Medicine & Rehabilitation, University of California, Irvine, CA, USA

**Keywords:** Stroke, Axon, Plasticity, Recovery, Treatment, Translation

## Abstract

Myelin-associated glycoprotein (MAG) is an inhibitor of axon growth. MAG levels increase after stroke. GSK249320 is a monoclonal antibody that neutralizes MAG-mediated inhibition and so may promote axon outgrowth and improve post-stroke outcomes. The current study tested the hypothesis that GSK249320 initiated 24 hours or 7 days after experimental stroke improves behavioural outcomes. Rats with right middle cerebral artery occlusion for 90 minutes were randomized to receive 6 weeks of intravenous (a) GSK249320 starting 24 hours post-stroke, (b) GSK249320 starting 7 days post-stroke, or (c) vehicle. Behavioral testing was performed over 7 weeks. Serial MRI demonstrated no differences in infarct volume across groups. Animals treated with GSK249320 24 hours post-stroke showed larger increases in Neuroscore (time X group, p = 0.0008) and staircase test (main effect of group, p = 0.0214) as compared to controls, but animals treated 7 days post-stroke showed no significant behavioral benefit. No significant results were found for the sticky tape or cylinder tests. A separate set of animals with experimental stroke received a single intravenous dose of GSK249320 or vehicle at 1 hour, 24 hours, 48 hours or 1 week post-stroke, and immunohistochemistry methods were used to measure GSK249320 distribution; GSK249320 was found in the ipsilesional hemisphere only, the extent of which increased with later times of injection. These data suggest that intravenous GSK249320 penetrates the lesion site and is associated with a small effect on functional outcomes when initiated 24 hours post-stroke and so support the translational potential of this monoclonal antibody as a restorative therapy for patients with stroke.

## Introduction

There are no approved therapies for improving deficits post-stroke that are initiated beyond the first few hours. Furthermore, in most studies where patients do receive an approved acute stroke therapy, most patients are left with significant deficits despite overall efficacy. Therapies based on brain repair have the potential to improve outcomes with a relatively wide time window.

One class of repair-based therapy revolves around promoting axon outgrowth to augment neuroplasticity. Such therapies target myelin-associated axon outgrowth inhibition molecules, which promote a non-permissive growth environment after stroke and so reduce neurite outgrowth [1–3]. Three major inhibitors of axon growth have been identified in myelin: oligodendrocyte-myelin glycoprotein, Nogo-A, and myelin-associated glycoprotein (MAG). Each is located in the periaxonal surface of the myelin membrane, binds to the Nogo-66 neuronal receptor (NgR), and activates the small GTPase Rho-A [4, 5]. Several studies, using divergent approaches, indicate that blockade of MAG promotes axonal growth and can improve recovery [5–11].

After stroke, MAG expression increases [12], further highlighting the potential to target post-stroke molecular events in order to promote neuroplasticity. GSK249320 is an IgG1-type humanized monoclonal antibody that neutralizes MAG-mediated inhibition [10]. A theoretical concern for an anti-MAG antibody is the potential to cause a demyelinating peripheral neuropathy, however, the Fc region of GSK249320 is disabled, preventing the complement fixation that is central to the pathophysiology of anti-MAG antibody neuropathy [13], and furthermore the anti-MAG antibodies associated with neuropathy are IgM not IgG [14].

The current study tested the hypothesis that GSK249320, an anti-MAG monoclonal antibody, when initiated 24 hours or later after induction of an experimental stroke in rodents, would be an effective restorative therapy, improving long-term behavioural outcomes without modifying infarct volume. Regarding choice of behavioural outcomes, the current focus is on the motor system, which is commonly affected in human subjects with stroke and which contributes substantially to disability. A secondary aim was to examine penetration of GSK249320 into the brain in rodents with an experimental stroke.

## Materials and Methods

### Experimental infarct

Surgery was performed on 66 male Sprague Dawley rats (Charles River, UK; weight 361 ± 21g, mean ± sd) under isoflurane anesthesia (in O_2_: medical air 20:80) by occluding the right middle cerebral artery (MCA) for 90 minutes with intraluminal thread (silicone rubber coated 5.0 monofilaments, tip diameter 0.33 ± 0.02 mm, from Doccol Corporation, USA) as previously described [15]. A total of 16 rats were excluded from the study because of sub-arachnoid hemorrhage (confirmed by MRI at 24 hour post-surgery, n = 8), because they died overnight (n = 3), or because were euthanized the day after surgery (n = 3) or at a later date (n = 2) due to excessively severe symptoms as required by the Home Office Project Licence. At the end of the experiment, on day 63 after middle cerebral artery occlusion (MCAO), animals were killed by transcardial perfusion with 0.9% saline, followed by 4% paraformaldehyde. All models used in these studies conformed to UK standards of animal care as laid down by the Home Office.

### Treatment

Rats were assigned to treatment groups using randomized block design; the blocks were stratified according to weight and animals within each block randomly assigned to one of three treatment groups. Animals then received IV injections through a tail vein of GSK249320 at 10 mg/kg starting 24 hours post-stroke (“MAG 24 hour”) and continuing weekly for 6 more doses (n = 14), GSK249320 at 10 mg/kg starting seven days post-stroke (“MAG 7d”) and continuing weekly for 5 more doses (n = 19; note that these animals also received an IV injection of vehicle 24 hours after MCAO), or vehicle starting 24 hours post-stroke (“VEH”) and continuing weekly for 6 more doses (n = 17). Animals were weighed at baseline then weekly.

The monoclonal antibody (GSK249320, molecular weight of 151.3 kDa) was received from GSK and diluted to 10 mg/ml before use. Solutions were stored at 4 °C. GSK249320 is expressed as a soluble glycoprotein secreted from a recombinant Chinese Hamster Ovary-DG44 cell line.

### Behavioral assessments

A multi-part motor scale and four functional tests were assessed in the left (contralesional) paw.

### The Neuroscore

This 9 part neurological assessment was assessed using modifications of Hunter et al. [16] and Modo et al. [17]. This battery assesses spontaneous motility, grooming, righting reflex, ability to grip a horizontal bar, visual paw placement, spontaneous and induced (via tail lift) circling, forelimb flexion, and contralateral reflex (lateral push). Maximum score is 18. Scoring was performed once before surgery, daily for the first seven days after MCAO, and then once/week thereafter.

### The staircase test

This test was performed as described by Virley et al. [15], using coco-pop pellets (Tesco) as food bait. This was performed 2, 4 and 6 weeks after MCAO. Before each test session the rats were placed on a mild food restriction diet comprising of 15g food pellets per rat (previously determined to be needed to maintain rats at 80–85% of free feeding weight). Prior to this, animals were trained each weekday for 3–4 weeks before surgery until they satisfied inclusion criteria of retrieval of at least 2 coco-pops, and displacement of less than 7 coco-pops, per side per day for 3 consecutive days. Performance was scored as the number of coco-pops recovered and/or displaced from each side. Each data point represents the mean of two trials per day over three days of testing per time-point. The last three training days pre-surgery, after the animal satisfied inclusion criterion, were used as the baseline score.

### The sticky tape test of sensorimotor neglect

For the sticky tape test [18] a 1 cm-wide strip of sticky tape (Micropore, 3M) was firmly wrapped around each forepaw. The latency to remove the tape was recorded in two trials per test point, each trial lasting up to three minutes. This test was conducted at 1, 3, 5 and 7 weeks after MCAO. Each data point represents a mean of two trials. Prior to MCAO surgery, animals were trained in four such sessions; the fifth session (1–3 days before MCAO) was used as the baseline measure.

### The cylinder test of forelimb-use asymmetry

The cylinder test [18] was conducted by placing rats inside a glass beaker (28.5 cm height and 13 cm base diameter) for up to 3 minutes, and the number of contacts between the glass wall and each forepaw was counted. This test was performed at 1, 3, 5 and 7 weeks after MCAO. Each test point comprised two such trials and each data point represents a mean of the two trials.

The sticky tape test of sensorimotor neglect [18] and the cylinder test of forelimb-use asymmetry [18] were conducted at 1, 3, 5 and 7 weeks after MCAO.

### Brain imaging

An MRI of the brain including T2-weighted anatomical images (TR = 4000 ms, TE = 60 ms, 45 x 0.6 mm thick slices with 0.31 x 0.31 mm in-plane resolution) was acquired at 24 hour post-stroke (prior to treatment in any group), and at the end of the experiment 63 days post-stroke. Lesion topography was determined from the first MRI (24 hour post-stroke), with infarcts classified as either (a) full MCA territory, i.e., involving cortical and subcortical regions, or (b) subcortical only, generally involving the striatum. Infarct volume was measured using DispImage software (D. Plummer, UCL) by contouring the lesion outline that remained after images were thresholded above 2SD of the MR signal intensity in the unaffected contralateral hemisphere. Brain volumes were obtained by automatic contouring.

### GSK249320 entry into the brain after stroke

A separate set of rats underwent MCAO as above. Animals were injected IV with either vehicle or a single dose of GSK249320 at a concentration of 10 mg/kg, at either 1 hour, 24 hours, 48 hours or 1 week following MCAO (n = 6 in each group). In order to determine antibody distribution in the brain, animals were killed by transcardiac perfusion with ice-cold saline, followed by 4% paraformaledehyde. Brains were stored in ice-cold 4% paraformaledehyde for 48 hours, then processed for paraffin embedding and subsequent histological analysis. Lesioned areas were delineated as areas of tissue with reduced haematoxylin and eosin.

In order to assess GSK249320 in these brains, paraffin embedded brain sections were cut at 4 μm, de-waxed, and hydrated into distilled water. An antigen retrieval method using Proteinase K (Dako:S3020) was applied to the sections for 5 minutes, after which slides were washed well in distilled water. Using a DAKO autostainer machine, Donkey anti-Human IgG (1:500, Jackson: 709-035-149) was applied to sections for 30 minutes, followed by an appropriate biotinylated anti-donkey IgG (1:200, Abcam: AB6764) for 30 minutes. Visualisation was accomplished using Vector ABC and DAB systems. Sections were then dehydrated, cleared and mounted.

### Statistical analysis

All data analysis used repeated measures or n-way ANOVA, with Fisher LSD post-hoc tests used to determine pairwise differences between experimental groups. Where terms for time or lesion topography were included in the model, comparisons between experimental groups were made at the highest level interaction term. All analyses were performed, and graphs produced, in Statistica 8.0 (StatSoft). Significance was defined at p<0.05. Where necessary, data were transformed prior to analysis in order to satisfy the statistical assumption of constant variance across the range of the response.

## Results

### Weight

The compound did not affect animals’ weight, and no significant difference in weight between groups was found, including when lesion topography was taken into consideration.

### Imaging

Infarct volumes decreased over time (from 256.9 ± 16 mm^3^ at 24 hour post-MCAO to 119.5 ± 6 mm^3^ at 46 days post-MCAO, mean ± SEM). There were no significant differences between the three treatment groups at either time point or over time. Also, there were no differences in brain volumes over time or between experimental groups. Lesion topography classification as determined from the 24 hour post-MCAO MRI found that the MAG 24 hour group had 7 full (cortical and subcortical) and 7 subcortical only strokes, the MAG 7d group had 11 full and 8 subcortical strokes, and the VEH group had 8 full and 9 subcortical strokes.

### Neuroscore

**Figure 1:**
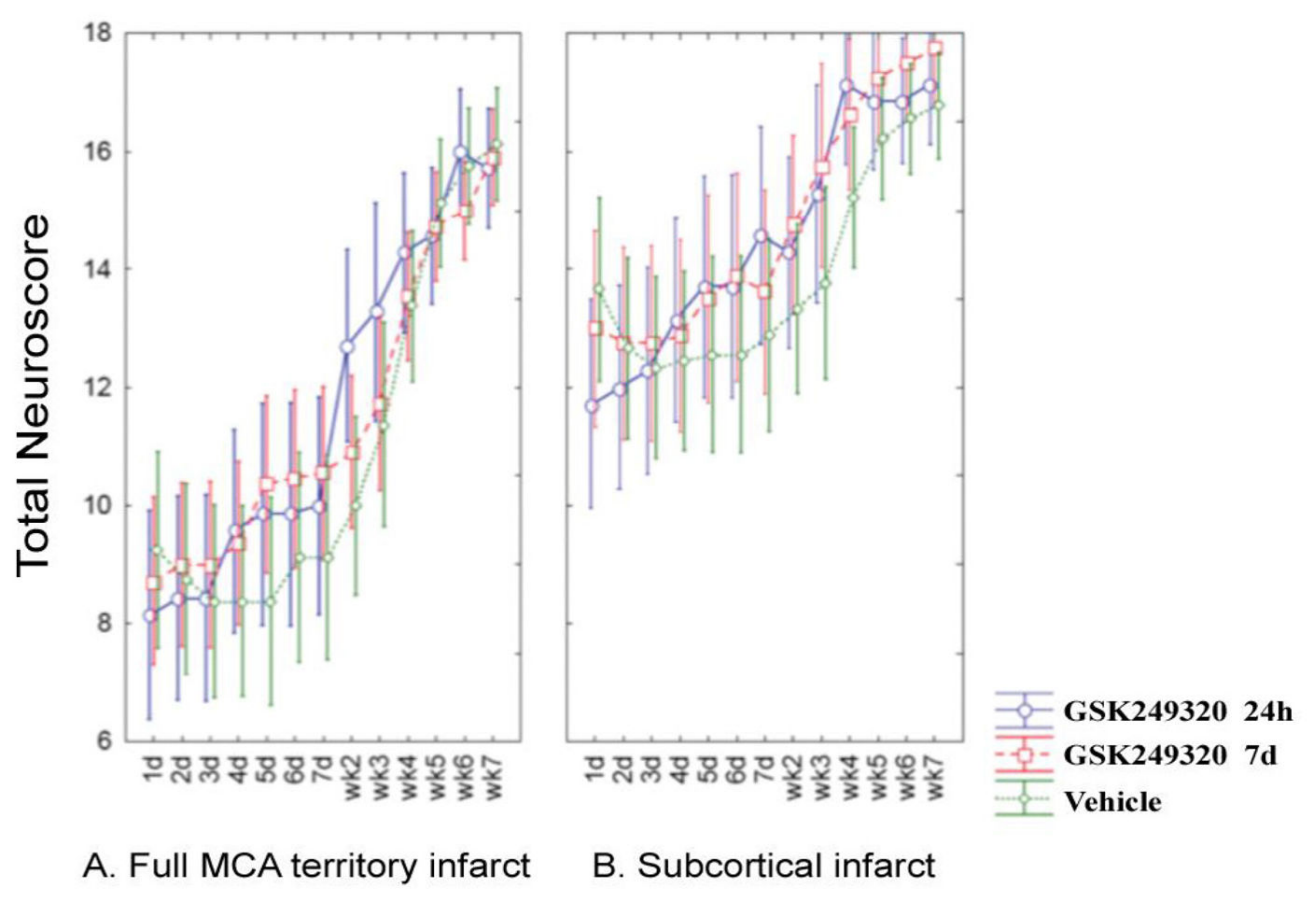
Change in Neuroscore over time according to stroke subtype. Mean values are plotted with 95% confidence intervals. [A] Data from animals with a full MCA territory infarct. [B] Data from animals with a subcortical infarct.

There was a significant interaction term in the repeated measures ANOVA between time and treatment group (p = 0.0008) that was not dependent on lesion topography (Figure 1). Post-hoc testing revealed significant (p<0.05) increases in the Neuroscore for MAG 24 hour vs VEH, on weeks 2 and 4 post-MCAO.

### Staircase test

**Figure 2:**
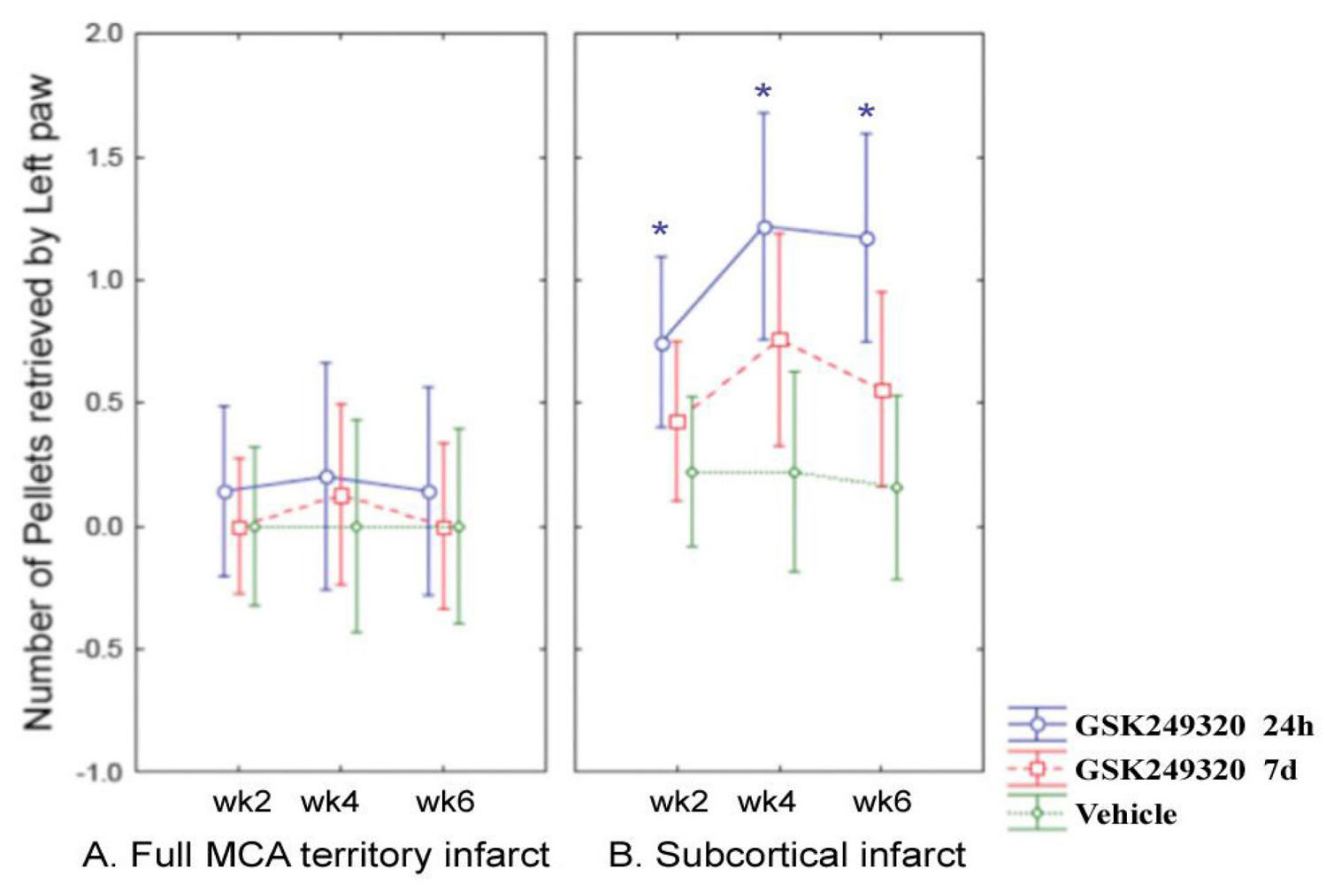
Change in staircase test over time according to stroke subtype (square root transform). Mean values are plotted with 95% confidence intervals. [A] Data from animals with a full MCA territory infarct. [B] Data from animals with a subcortical infarct. *specific timepoints where a significant increase was seen in the number of pellets retrieved for MAG 24h animals (subcortical infarct group) as compared to VEH in post hoc testing.

Repeated measures ANOVA found the main effect of treatment group to be significant, in the group of animals with subcortical lesions only (p = 0.0214, Figure 2). Post-hoc testing revealed a significant increase in the number of pellets retrieved for MAG 24 hour animals as compared to VEH on weeks 2, 4, and 6 in this group (p = 0.0261, 0.0021, and 0.0007, respectively).

### Sticky tape test

**Figure 3:**
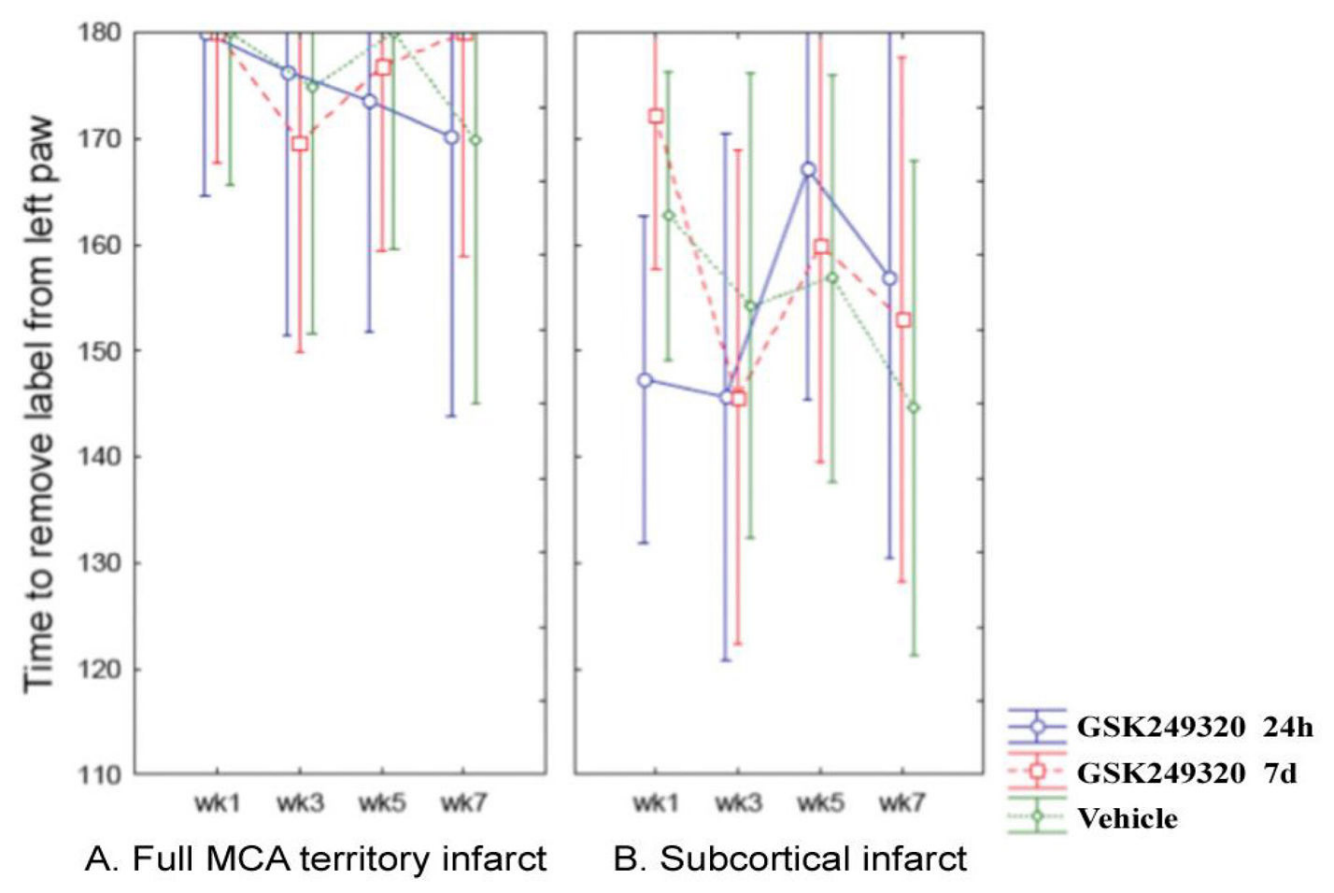
Change in sticky tape test over time according to stroke subtype. Mean values are plotted with 95% confidence intervals. [A] Data from animals with a full MCA territory infarct. [B] Data from animals with a subcortical infarct.

There were no significant differences between treatments groups over time (Figure 3).

### Cylinder test

**Figure 4:**
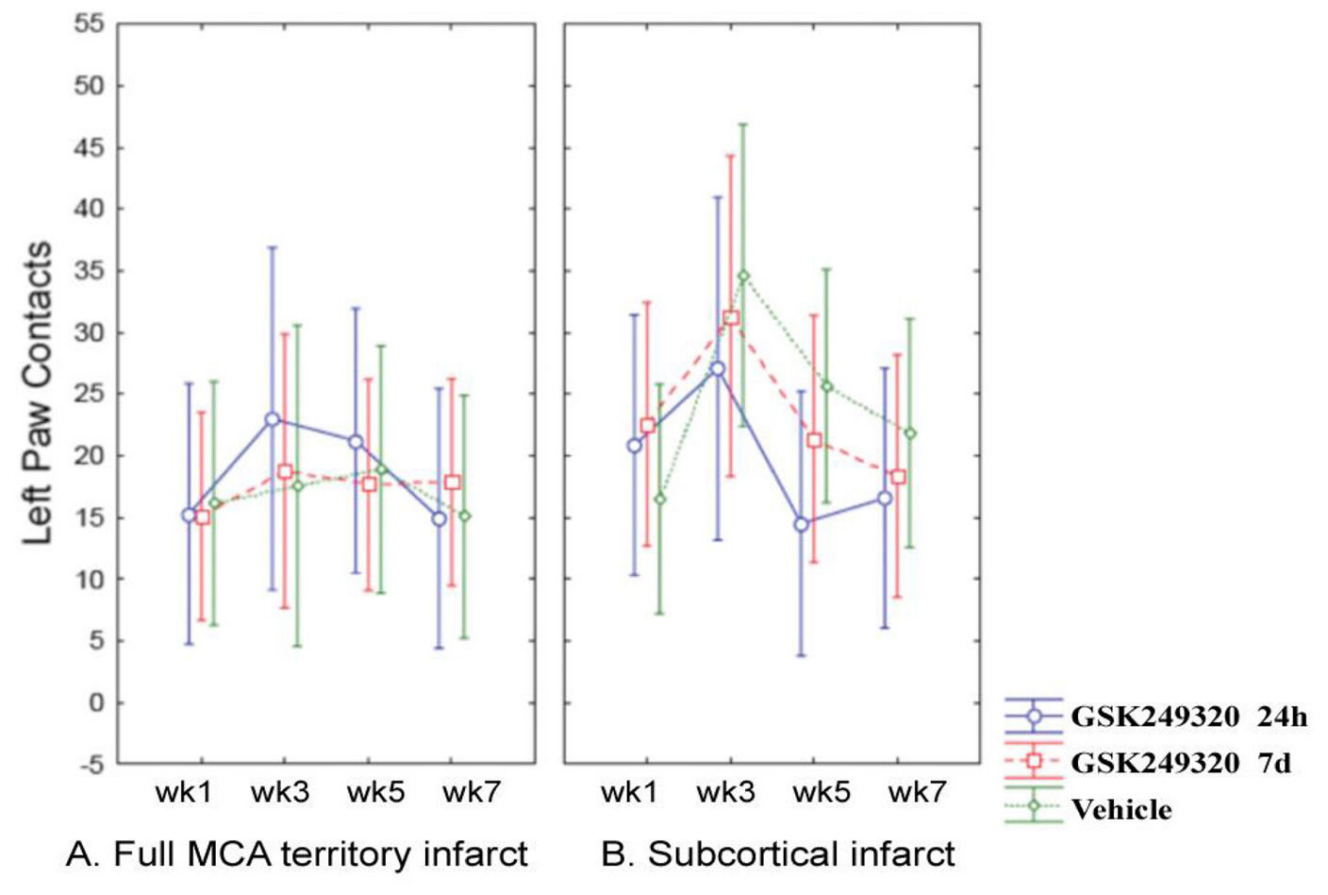
Change in cylinder test over time according to stroke subtype. Mean values are plotted with 95% confidence intervals. [A] Data from animals with a full MCA territory infarct. [B] Data from animals with a subcortical infarct.

There were again no significant differences between treatments groups over time (Figure 4).

### GSK249320 entry into the brain after stroke

**Figure 5:**
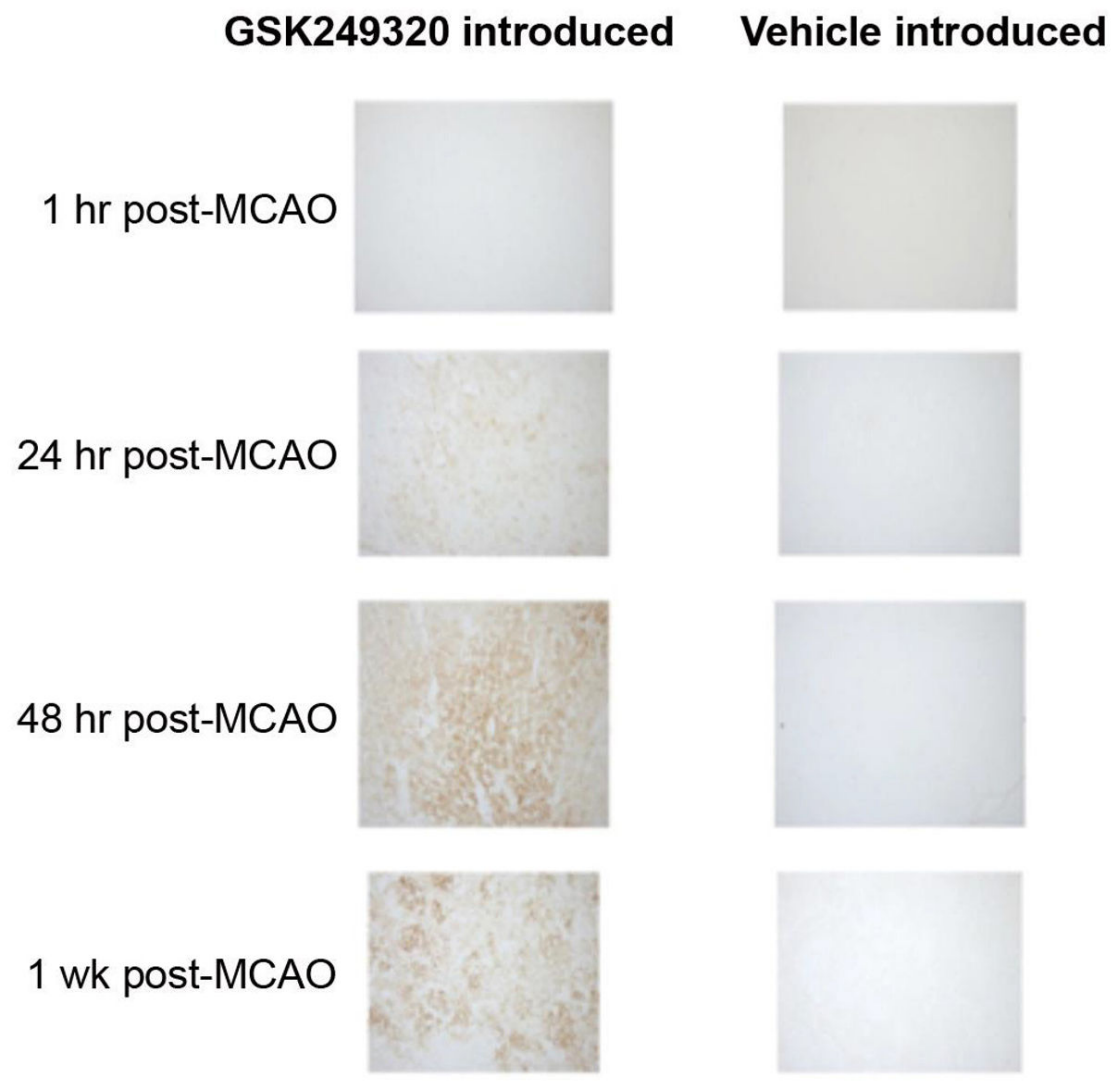
Staining for GSK249320 in the ipsilesional hemisphere.

At all four post-MCAO time points examined, administration of GSK249320, followed by sacrifice and perfusion 6 hours later, revealed staining for GSK249320 in the ipsilesional, but not contralesional, hemisphere, and no staining was seen in either brain hemisphere following vehicle administration (Figure 5). This ipsilesional hemisphere staining 6 hours following IV GSK249320 administration was light when GSK249320 was introduced 1 hour post-MCAO, moderate when GSK249320 was introduced at 24 hours post-MCAO, strong when GSK249320 was introduced at 48 hours post-MCAO, and strongest when GSK249320 was introduced at 7 days post-MCAO.

## Discussion

Restorative therapies aim to promote repair after stroke and so complement neuroprotective and reperfusion therapies given in the initial hours after stroke, which aim to reduce injury. Consistent with this, GSK249320, a restorative therapy that aims to improve outcomes by blockage of MAG, penetrated lesion sites and improved behavioral outcomes without having an effect on infarct volume. Effects were most pronounced in animals receiving GSK249320 beginning 24 hours post-MCAO. Significant effects were found for two of the four measures, Neuroscore and staircase test, although this must be considered a small effect given that findings were significant only at some of the time points, and for the staircase test only in animals with a subcortical infarct. Data confirm that GSK249320 is able to enter the brain at this time post-injury. Together these results support the potential utility of GSK249320 as a restorative therapy in the treatment of stroke.

These studies examined the ability of GSK249320, a monoclonal antibody against MAG, to improve functional outcomes in a rat model of ischemic stroke. MAG expression inhibits neurite outgrowth and therefore limits CNS regeneration. MAG expression increases after stroke [12], further increasing the potential importance of this molecule as a therapeutic target. Prior studies support the hypothesis that blockade of MAG promotes axonal growth: introduction of a dominant-negative form of NgR in rodents substantially reduces the inhibitory effects of MAG, MAG inhibition of developing cerebellar or dorsal root ganglia neurite outgrowth is reversed in vitro by an anti-MAG antibody, immunodepletion of MAG from bovine CNS myelin substantially diminishes its neurite outgrowth inhibitory activity, and inactivation of the small guanosine triphosphatase (GTPase) Rho-A as well as its downstream effector Rho-associated serine-threonine kinase reduces MAG-mediated neurite outgrowth inhibition [5, 6, 9]. GSK249320, as a therapy designed to neutralize MAG, aimed to build on these observations and was hypothesized to increase clinically favorable neural plasticity and promote improved outcomes after brain injury such as stroke.

The current results support this hypothesis. The Neuroscore showed benefit from GSK249320 (Figure 1) that appeared to begin emerging within days of first administration, in both cortical strokes and subcortical strokes, and this was most significant in the group receiving the antibody 24 hours post-stroke. This scale assesses a range of motor-related behaviors such as motility, righting, paw placement, and circling, and so provides a rationale for translational studies that focus on motor-related behaviors. The staircase test (Figure 2) found benefit from GSK249320, but only in animals with subcortical stroke, and this was significant in the group receiving the antibody 24 hours post-stroke. One reason for a greater effect in animals with a subcortical infarct may be a floor effect of the staircase test in animals with severe brain injury. This might also suggest that any translational studies of GSK249320 could have greater effect in patients with strictly subcortical stroke, which constitute 50 [19] to 66% [20] of all ischemic strokes, although this consideration must be viewed with caution given the limited sample sizes in the current study. GSK249320 was not superior to control on the simple tests of asymmetry and basic limb use, possibly suggesting broad effects on complex motor networks. Histological studies found that GSK249320 entered the ipsilesional hemisphere as early as 1 hour after MCAO (Figure 5), with increasing antibody ingress over time. Together these results suggest that blockade of MAG is an effective strategy to access brain tissue surviving an infarct, where the antibody has the effect of improving behavioral outcomes, that this effect is superior with earlier exposure, and that there may be some advantage when the infarct is subcortical. The finding that some behavioral measures were positive and others were not, that any significant effects were present only on some of the days examined, and that results were significant for only subcortical stroke for one of the measures indicate that behavioral effects while significant were small.

GSK249320 entered the ipsilesional hemisphere at all time points examined, being greater when introduced 7 days post-stroke as compared to 24 hours post-stroke. Antibodies normally have low efficiency to cross the blood-brain barrier (BBB) [21], and so the finding that GSK249320 accumulated preferentially within the ipsilesional hemisphere over time supports the idea that it may have entered the brain more efficiently due to defects in BBB integrity created by the stroke. Although ipsilesional staining was maximal when GSK249320 was introduced 7 days after stroke, significant behavioral gains were only seen when GSK249320 was initiated 24 hours post-stroke. Antibody entry alone may therefore be insufficient to promote neural repair and improve behavioral outcomes. A number of genetic, physiological, and biochemical events are in flux between days 1 and 7 post-stroke [12, 22–24]. The exact factors that might have mediated the differences in GSK249320 effects according to time of administration cannot be determined in the current study, however, it is noted that current findings are consistent with prior studies that administered growth factors to rats at varying times post-stroke. For example, Ren et al. [25] found that initiating osteogenic protein-1 to rats with experimental infarct 1 or 3 days, but not 7 days, post-stroke improved motor behavioral outcomes, and Kolb et al. [26] found that sequential growth factors (epidermal growth factor followed by erythropoietin) initiated 0 or 3 days post-stroke showed greater behavioral gains compared to when therapy was initiated 7 days post-stroke. These results emphasize the importance of time post-stroke in the design of restorative therapy protocols for this disease setting.

Prior studies in rodents found that after CNS injury, an antibody against MAG can neutralize MAG-mediated inhibition, promote neuritic outgrowth, and improve behavioral recovery [10, 11]. The current study builds on these findings, providing evidence that GSK249320, an anti-MAG monoclonal antibody, initiated IV 24 hours after stroke onset enters the ipsilesional hemisphere and is associated with a small effect on functional outcomes. This study was conducted in a manner concordant with STAIR criteria [27] and so supports the translational potential of this monoclonal antibody as a restorative therapy in patients with stroke.

## Conclusion

Intravenous GSK249320 penetrates the lesion site and is associated with a small effect on functional outcomes when initiated 24 hours post-stroke

## Abbreviations

MAG: Myelin-associated glycoprotein
NgR: Nogo-66 neuronal receptor
MCAO: Middle cerebral artery occlusion

## References

[1] Schwab ME, Strittmatter SM (2014). Nogo limits neural plasticity and recovery from injury. Curr Opin Neurobiol.

[2] Fang PC, Barbay S, Plautz EJ, Hoover E, Strittmatter SM (2010). Combination of NEP 1–40 treatment and motor training enhances behavioral recovery after a focal cortical infarct in rats. Stroke.

[3] Lee JK, Kim JE, Sivula M, Strittmatter SM (2004). Nogo receptor antagonism promotes stroke recovery by enhancing axonal plasticity. J Neurosci.

[4] GrandPre T, Nakamura F, Vartanian T, Strittmatter SM (2000). Identification of the Nogo inhibitor of axon regeneration as a Reticulon protein. Nature.

[5] Walmsley AR, Mir AK (2007). Targeting the Nogo-A signalling pathway to promote recovery following acute CNS injury. Curr Pharm Des.

[6] Mukhopadhyay G, Doherty P, Walsh FS, Crocker PR, Filbin MT (1994). A novel role for myelin-associated glycoprotein as an inhibitor of axonal regeneration. Neuron.

[7] Torigoe K, Lundborg G (1998). Selective inhibition of early axonal regeneration by myelin-associated glycoprotein. Exp Neurol.

[8] Mears S, Schachner M, Brushart TM (2003). Antibodies to myelin-associated glycoprotein accelerate preferential motor reinnervation. J Peripher Nerv Syst.

[9] Domeniconi M, Filbin MT (2005). Overcoming inhibitors in myelin to promote axonal regeneration. J Neurol Sci.

[10] Irving EA, Vinson M, Rosin C, Roberts JC, Chapman DM (2005). Identification of neuroprotective properties of anti-MAG antibody: a novel approach for the treatment of stroke?. J Cereb Blood Flow Metab.

[11] Thompson HJ, Marklund N, LeBold DG, Morales DM, Keck CA (2006). Tissue sparing and functional recovery following experimental traumatic brain injury is provided by treatment with an anti-myelin-associated glycoprotein antibody. Eur J Neurosci.

[12] Li S, Carmichael ST (2006). Growth-associated gene and protein expression in the region of axonal sprouting in the aged brain after stroke. Neurobiol Dis.

[13] Latov N, Renaud S (2004). Effector mechanisms in anti-MAG antibody-mediated and other demyelinating neuropathies. J Neurol Sci.

[14] Steck AJ, Stalder AK, Renaud S (2006). Anti-myelin-associated glycoprotein neuropathy. Curr Opin Neurol.

[15] Virley D, Beech JS, Smart SC, Williams SC, Hodges H (2000). A temporal MRI assessment of neuropathology after transient middle cerebral artery occlusion in the rat: correlations with behavior. J Cereb Blood Flow Metab.

[16] Hunter AJ, Hatcher J, Virley D, Nelson P, Irving E (2000). Functional assessments in mice and rats after focal stroke. Neuropharmacology.

[17] Modo M, Stroemer RP, Tang E, Veizovic T, Sowniski P (2000). Neurological sequelae and long-term behavioural assessment of rats with transient middle cerebral artery occlusion. J Neurosci Methods.

[18] Schallert T, Fleming SM, Leasure JL, Tillerson JL, Bland ST (2000). CNS plasticity and assessment of forelimb sensorimotor outcome in unilateral rat models of stroke, cortical ablation, parkinsonism and spinal cord injury. Neuropharmacology.

[19] Kang DW, Chalela JA, Ezzeddine MA, Warach S (2003). Association of ischemic lesion patterns on early diffusion-weighted imaging with TOAST stroke subtypes. Arch Neurol.

[20] Wessels T, Wessels C, Ellsiepen A, Reuter I, Trittmacher S (2006). Contribution of diffusion-weighted imaging in determination of stroke etiology. AJNR Am J Neuroradiol.

[21] Paul SM (2011). Therapeutic antibodies for brain disorders. Sci Transl Med.

[22] Murphy TH, Corbett D (2009). Plasticity during stroke recovery: from synapse to behaviour. Nat Rev Neurosci.

[23] Nudo RJ (2007). Postinfarct cortical plasticity and behavioral recovery. Stroke.

[24] Hermann DM, Chopp M (2012). Promoting brain remodelling and plasticity for stroke recovery: therapeutic promise and potential pitfalls of clinical translation. Lancet Neurol.

[25] Ren J, Kaplan P, Charette M, Speller H, Finklestein S (2000). Time window of intracisternal osteogenic protein-1 in enhancing functional recovery after stroke. Neuropharmacology.

[26] Kolb B, Morshead C, Gonzalez C, Kim M, Gregg C (2007). Growth factor-stimulated generation of new cortical tissue and functional recovery after stroke damage to the motor cortex of rats. J Cereb Blood Flow Metab.

[27] Stroke Therapy Academic Industry Roundtable (STAIR) (1999). Recommendations for standards regarding preclinical neuroprotective and restorative drug development. Stroke.

